# Around the Hospitals

**Published:** 1895-10-12

**Authors:** 


### AROUND THE HOSPITALS.

[Notice to the Managers of Institutions.—Special space is now reserved for the insertion of notices of hospital meetings and festivals. The Editor requests that all notices may reach The Hospital Office, 428, Strand, at least one week before the date of each meeting.]

**The Sunderland Infirmary.**—The work at this infirmary has been well maintained during the past year. There have not been quite so large a number of patients in the wards, but those received have remained longer than in the previous year. There is a slight diminution visible on the receipts from nearly all sources, but the management do not express themselves dissatisfied, as bad times can be pleaded to explain the deficiencies. The expenditure shows a diminution, and the cost of maintaining the institution may be regarded as low, amounting as it does to £42 17s. 2d. per occupied bed. Although in keeping accounts the uniform system is not adopted, the particulars given of the items of expenditure are so full that the usual difficulty in forming a just comparison where the system is absent is avoided. The report should prove very useful in the secretariat of many institutions. The expenditure on wines and spirits is remarkably small, being fourpence per patient. This, of course, is a point rather for the medical staff than the management, but in any case one that might be noted with advantage. It is proposed to extend the accommodation for the reception of patients applying for admission by an expenditure of £150. Shelters in the grounds have been added, and are much appreciated. We note with interest that in the list of bequests the fund for pensioning nurses, in connection with the hospital, has been added to. This fund now reaches a sum of £1,488. We believe that the infirmary would make a better arrangement for the advantage of their nurses by affiliating with the Royal National Pension Fund for Nurses. The Heatherdene Convalescent Home is doing useful service, and the building of the new wing is proceeding, but cannot be finished owing to the need of £850 to complete the necesary funds. During the year 2,362 in-patients and 4,853 out-patients were received in the infirmary. The number of out-patients shows an increase of 1,560. The ordinary income amounted to £8,435, and the ordinary expenditure to £8,451. The cost per patient was £3 7s. 5d.

**Royal Albert Edward Infirmary, Wigan.**—We would firstly draw attention to the excellently drawn up report issued by the institution. It is full of explicit information, so arranged as to be interesting and most easy of comprehension. The side headings adopted by Mr. Taberner, the secretary, are very useful; we wish that more institutions would carry out the same method. A large number of improvements have been carried out at the infirmary during the past year. These include enlarged dining accommodation for the staff and other alterations estimated to cost £3,000. The heating apparatus has been extended at a cost of upwards of £430, and a new mortuary has been acquired. We are glad to note that the expenditure of the infirmary, though still high for a provincial institution, shows some reduction in the average cost per occupied bed. Last year this amounted to £63 6s. 11d., as against £64 12s. 6d. in the previous year. Mr. Taberner has not adopted the uniform system of accounts, or doubtless he would find it easy to place his finger upon the weak spots in expenditure when compared with institutions similar to his own. When writing our notes upon metropolitan hospitals we have frequently pointed out the necessity of further convalescent aid in connection with them, such being so obviously wanted and of the utmost importance. The study of convalescent funds attached to provincial hospitals seems to show that this is not the case in the provinces. Frequently we have observed a trivial expenditure, and a continuously large balance in favour of the fund. So it is with Wigan. The whole expenditure for the year amounted to but £29 10s., leaving a balance of £122 13s. In such a district as Wigan, it seems strange that more help is not required than £130 can supply. In winter surely, if the fund were regarded as benevolent as well as convalescent, a great deal could be done in supplying warm clothing when such is needed to outgoing patients. The number of patients attending the infirmary is large. Last year 1,524 in-patients and 6,958 out-patients were treated. The ordinary expenditure amounted to £7,289, and the income to £7,769, which included sums received for the building fund and the convalescent fund.

